# Multiple conversion between the genes encoding bacterial class-I release factors

**DOI:** 10.1038/srep12406

**Published:** 2015-08-10

**Authors:** Sohta A. Ishikawa, Ryoma Kamikawa, Yuji Inagaki

**Affiliations:** 1 Graduate School of Life and Environmental Sciences, University of Tsukuba, Tsukuba, Ibaraki, Japan; 2 Center for Computational Sciences, University of Tsukuba, Tsukuba, Ibaraki, Japan; 3 Graduate School of Human and Environmental Studies, Kyoto University, Kyoto, Kyoto, Japan; 4 Graduate School of Global Environmental Studies, Kyoto University, Kyoto, Kyoto, Japan

## Abstract

Bacteria require two class-I release factors, RF1 and RF2, that recognize stop codons and promote peptide release from the ribosome. RF1 and RF2 were most likely established through gene duplication followed by altering their stop codon specificities in the common ancestor of extant bacteria. This scenario expects that the two RF gene families have taken independent evolutionary trajectories after the ancestral gene duplication event. However, we here report two independent cases of conversion between *RF1* and *RF2* genes (*RF1*-*RF2* gene conversion), which were severely examined by procedures incorporating the maximum-likelihood phylogenetic method. In both cases, *RF1*-*RF2* gene conversion was predicted to occur in the region encoding nearly entire domain 3, of which functions are common between RF paralogues. Nevertheless, the ‘direction’ of gene conversion appeared to be opposite from one another—from *RF2* gene to *RF1* gene in one case, while from *RF1* gene to *RF2* gene in the other. The two cases of *RF1-RF2* gene conversion prompt us to propose two novel aspects in the evolution of bacterial class-I release factors: (i) domain 3 is interchangeable between RF paralogues, and (ii) *RF1-RF2* gene conversion have occurred frequently in bacterial genome evolution.

During translation in Bacteria, Archaea, and Eukarya, codons in mRNAs are recognized by aminoacyl tRNAs with cognate anticodons, except three stop codons. Three stop codons in the standard genetic code, namely UAA, UGA, and UAG, are recognized by class-I release factors (RFs)^1^,^2^,^3^,^4^. In Eukarya and Archaea, a single class-I RF recognizes all of the three stop codons and terminates peptide synthesis^5^,^6^. Bacteria (and bacterium-derived organelles in eukaryotes) requires two class-I RFs, RF1 and RF2, with distinct codon specificities; the former recognizes UAA and UAG, and the latter does UAA and UGA^6^,^7^. In addition, bacterial lineages (including the phylum Bacteroidetes assessed in this study; see below) possess a protein family, of which amino acid (aa) sequences bear clear similarity to RF1 and RF2, named RFH. Based on the sequence similarity between RFH and RF1/2, RFH was hypothesized to be involved in translation^8^.

In all bacteria with the standard genetic code, neither RF1 nor RF2 is dispensable due to their principal roles in translation. RF1 and RF2 were functionally diverged from one another to recognize different sets of stop codons, although the two proteins share the significant sequence similarity at the primary to tertiary structural levels^9^,^10^,^11^. These observations strongly suggest that two distinct class-I RFs emerged *via* gene duplication followed by functional divergence in the last common ancestor of bacteria^12^. At the tertiary structure level, RF1 and RF2 commonly comprise four domains^9^,^10^,^11^,^13^. Both RF1 and RF2 contains universally conserved three consecutive aa residues in domain 3, glycine-glycine-glutamine (GGQ motif), which promotes hydrolysis of the peptidyl-tRNA on the A site of the ribosome upon stop codon recognition. Domains 2 and 4 as a whole involve in stop codon recognition. RF1 domain 2 directly recognizes stop codons by consecutive aa residues proline-[any amino acid]-threonine (PXT motif). Reflecting the difference in codon specificity between RF paralogues, RF2 domain 2 possess ‘SP(F/Y)’ motif, comprising serine, proline, and phenylalanine or tyrosine, instead of PXT motif in RF1 domain 2. Domain 1 in both RF1 and RF2 are involved in neither stop codon recognition nor peptidyl hydrolysis, but may be a scaffold for class-II release factor (RF3), which binds to RF1 and RF2, and enhances the peptide-release activity upon hydrolysis of GTP^14^.

RF1 and RF2 were most likely separated from each other at a very early stage of bacterial evolution^12^. Nevertheless, we noticed that a characteristic sequence motif ‘shared’ between RF1 and RF2 in a member of the class Bacteroidia, *Bacteroides thetaiotaomicron*^8^. The aa sequence alignment presented in Baranov *et al.* (2006)^8^ displayed that *B. thetaiotaomicron* RF1 and RF2 ‘shared’ a motif of 12 aa residues in the homologous position, which were absent from any other RF sequences in the particular alignment (see Fig. 1 in the original article). As independent acquisition of the homologous motif in RF1 and RF2 is highly unlikely, there are two scenarios for the motif ‘shared’ exclusively between *B. thetaiotaomicron* RF1 and RF2. The first scenario assumes that the motif predates the separation of RF1 and RF2, followed by (potentially massive) parallel losses of the motif in both RF1 and RF2 of extant bacteria, except *B. thetaiotaomicron* (and its relatives; see below). Alternatively, either RF1 or RF2 may have acquired the motif in the common ancestor of a taxonomic unit including *B. thetaiotaomicron*, followed by a conversion of the RF gene fragment encoding a portion encompassing the motif into the paralogous gene, of which product lacked the motif.

From the textbook view, gene conversion homogenizes multiple gene copies, of which sequences are identical or nearly identical, in a genome. A mutation occurred in one of multiple gene copies, if it is deleterious, can be erased quickly by gene conversion between the mutated and original copies^15^,^16^,^17^. Gene conversion potentially works similar but in the opposite direction, if a mutation is advantageous. The beneficial mutation occurred in one of multiple gene copies can spread to other gene copies by gene conversion^18^,^19^,^20^. In addition to the ‘classical’ gene conversion between multiple gene copies described above, the ‘non-classical’ cases of gene conversion between evolutionarily distant sequences—orthologous sequences in different genomes and paralogous sequences in the same genome—were documented by analyzing bacterial^21^, archaeal^22^,^23^,^24^, and eukaryotic^25^,^26^,^27^ genomes in literature.

In this study, we investigated the conversion between *RF1* and *RF2* genes in the class Bacteroidia, which was provoked by a characteristic sequence motif shared between *B. thetaiotaomicron* RF1 and RF2. Prior to this study, sequence motifs (including insertion sequences) shared between distantly related sequences were considered as the signs of non-classical gene conversion^22^,^23^,^24^. Thus, the motif shared between RF paralogues hints a conversion between *RF1* and *RF2* genes during the evolution of Bacteroidia. To explore the potential conversion between *RF1* and *RF2* genes (*RF1-RF2* gene conversion), we investigated RF1 and RF2 sequences sampled from diverse members of Bacteroidia in this study. Systematic surveys of the phylogenetic signal of gene conversion, together with the phylogenetic distribution of the motif in Bacteroidia, consistently suggest a single non-classical gene conversion between *RF1* and *RF2* genes in the ancestral genome of Bacteroidia, followed by multiple motif ‘reversions’ *via* additional gene conversion events in several descendent lineages. In addition, our survey of RF1 and RF2 sequences sampled from diverse bacteria revealed the second case of *RF1-RF2* gene conversion in the evolution of the phylum Chroloflexi. The present study implies frequent *RF1-RF2* gene conversion after the divergence of bacterial lineages.

## Results and Discussion

### An early origin of ‘12 aa-motif’ in Bacteroidia

**Figure 1:**
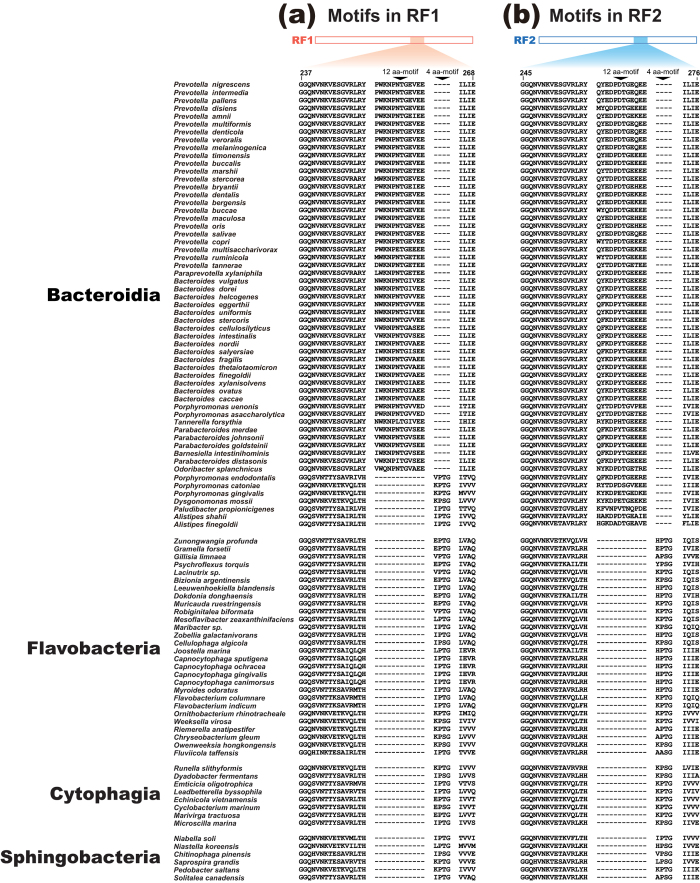
‘12 aa-motif’ and ‘4 aa-motif’ in RF paralogues of 99 members of Bacteroidetes. (**a**) RF1 amino acid (aa) alignment that corresponds to aa residues 237–268 in *Prevotella nigrescens* RF1 (GenBank accession no. EGQ17478.1). (**b**) RF2 aa alignment that corresponds to aa residues 245–276 in *P. nigrescens* RF2 (GenBank accession no. EGQ14454.1).

RF1 and RF2 sequences were split in both maximum-likelihood (ML) and Bayesian phylogenetic analyses of an alignment comprising 230 unambiguously aligned aa positions of the pairs of RF1 and RF2 sequences sampled from 99 members belonging to the phylum Bacteroidetes (Fig. S1). The split between RF paralogues were supported by a ML bootstrap support value (MLBP) of 100% and a Bayesian posterior probability (BPP) of 1.00 (Fig. S1). We anticipated the separation between RF1 and RF2 sequences, as the two paralogues are most likely established through a single gene duplication followed by divergence of codon specificity in the last common ancestor of extant bacteria^12^. Nevertheless, with respect to the ancient separation of the RF paralogues, it is unexpected to find that a unique motif of 12 aa in length (12 aa-motif) is shared between the RF paralogues sampled from the vast majority of the 57 members of the class Bacteroidia examined in this study (Fig. 1A,B; see below for the details). Curiously, 12 aa-motif was not observed in any RF paralogues of all bacterial lineages except Bacteroidia (data not shown).

**Figure 2:**
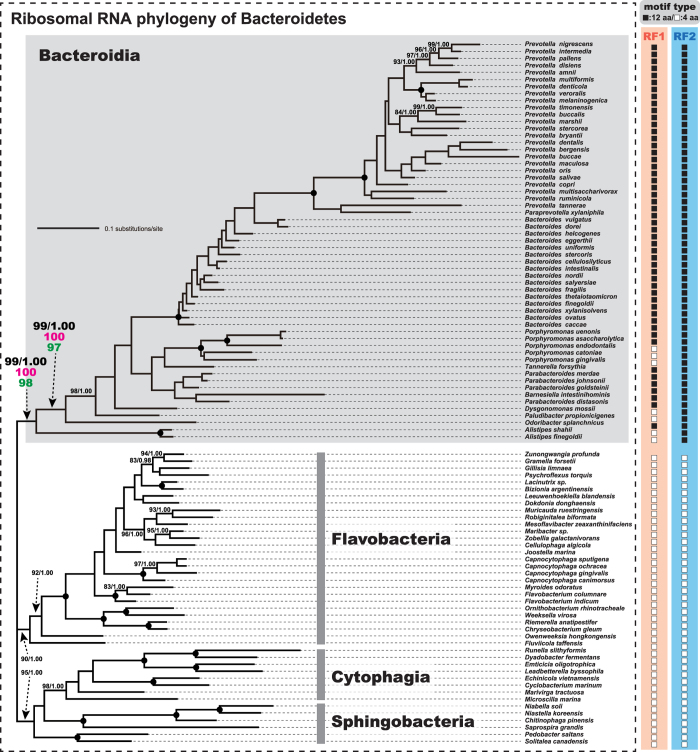
Organismal phylogeny of Bacteroidetes inferred from a concatenated 16S and 23S ribosomal RNA alignment. The final ribosomal RNA alignment comprising 3,279 unambiguously aligned nucleotide positions was subjected to the maximum-likelihood (ML) and Bayesian methods. As the two methods reconstructed very similar trees, only the ML tree is shown here. Only ML bootstrap support values (MLBPs) >80% and Bayesian posterior probability (BPPs) >0.90 are shown in this figure. Dots correspond to MLBP of 100% and BPP of 1.00. For the deepest and second deepest positions of *Alistipes* spp. and *Odoribactor splanchnicus* in the Bacteroidia clade (shaded), we present the MLBPs from the ML analyses of (i) the alignment processed by ‘RY-coding’ procedure (shown in magenta), and (ii) the alignment excluding the sequences of which G + C contents significantly depart from the average G + C content calculated from the 99 sequences in the original alignment (shown in green). On the right side of the rRNA tree, we schematically present the motif type in RF1 and RF2 for each taxon—12 aa-motif (closed boxes) or 4 aa-motif (open boxes).

It is intriguing how 12 aa-motif was shared between the anciently separated RF paralogues in a single bacterial class. We mapped the presence and absence of 12 aa-motif in RF1/RF2 onto the ML tree inferred from a concatenated alignment of 16S and 23S rRNA sequences (3,729 unambiguously aligned nucleotide positions in total), which represents the organismal relationship of the 99 taxa in Bacteroidetes (Fig. 2). As the overall tree topology reconstructed by Bayesian method was essentially identical to the ML tree, only BPPs of 1.00 were indicated in Fig. 2. The rRNA phylogeny successfully reconstructed the 57 members of Bacteroidia as monophyly with a MLBP of 99% and a BPP of 1.00. Among the 57 member of Bacteroidia, the branch leading to *Alistipes finegoldii* and *A. shahii*, and that leading to *Odoribacter splanchnicus* were defined as the earliest and second earliest branches, respectively, with high statistical support (MLBPs of 99% and BPPs of 1.00; Fig. 2). Additionally, we evaluated the potential impact of the variation in the content of guanine plus cytosine (G + C contents) across a tree by re-analyzing the rRNA alignments processed by (i) excluding the sequences of which G + C contents were significantly departed from the average G + C content calculated from the 99 sequences, and (ii) ‘RY-coding’ procedure^28^,^29^ (The details are described in the Methods). Although not shown here, the early branching pattern in the Bacteroidia clade, which is critical to infer how 12 aa-motif emerged in RF evolution, appeared to be unchanged in the second and third analyses, suggesting that our proposal for the timing of the emergence of 12 aa-motif in RF1/2 is unlikely affected by the variation in G + C content across a tree (see below for the details). Thus, we indicated the MLBP values from the second and third ML analyses for the two nodes mentioned above in Fig. 2 (highlighted in magenta and green), and provide the results from the second ML/ML bootstrap analyses as Fig. S2 for readers’ convenience.

In terms of the presence and absence of 12 aa-motif, the motif was found in the RF2s of all members of Bacteroidia examined here (closed squares in Fig. 2; see also Fig. 1B), suggesting that the common ancestor of the members of Bacteroidia had already had a RF2 with 12 aa-motif (12aa_motif-type RF2). On the other hand, the RF1s with 12 aa-motif (12aa_motif-type RF1) was not ubiquitous in the members of Bacteroidia examined here (Fig. 2). Out of the 57 members of Bacteroidia, seven members [*A. finegoldii*, *A. shahii*, *Paludibacter propionicigenes*, *Dysgonomonas mossii*, *Porphyromonas* (*Po*) *gingivalis*, *Po. cateniae*, and *Po. endodontalis*] were found to possess the RF1s with a sequence motif of four aa residues (open squares in Fig. 2; see also Fig. 1A), instead of 12 aa-motif. ‘4 aa-motif’ is seemingly ancestral to both RF1 and RF2, as RF paralogues of a phylogenetically broad spectrum of bacteria (including members in the classes of Flavobacteria, Cytophagia, and Sphigobacteria) possess this short motif rather than 12 aa-motif (Fig. 1A,B).

### Sliding window analyses detected the signal for conversion between *RF1* and *RF2* genes

There are a number of literatures reporting gene conversion among gene families that were emerged from gene duplications, regardless of their evolutionary distance^21^,^22^,^23^,^24^,^25^,^26^,^27^. We here assessed whether 12 aa-motif shared between RF paralogues of a restricted bacterial taxa (Bacteroidia) is the product of the conversion between two paralogous genes.

The possible conversion between *RF1* and *RF2* genes (*RF1-RF2* gene conversion) was examined by a sliding window (SW) analysis using the ML phylogenetic method^22^,^23^,^24^. From the original ‘RF’ alignment including the 99 pairs of RF1 and RF2 sequences (see above), we generated ‘6-pair’ alignments included (i) a single pair of RF1 and RF2 sequences with 12 aa-motif of a member of Bacteroidia, and (ii) those with 4 aa-motif of five randomly-selected species belonging to the class Flavobacteria or Cytophagia (*Psychroflexus torquis*, *Microscilla marina,* and *Riemerella anatipestifer*, *Gillisia limnaea* and *Emticicia oligotrophica*). As the original alignment contained the pairs of RF1 and RF2 sequences with 12 aa-motif of 50 members of Bacteroidia, we generated and analyzed 50 different 6-pair alignments in this study. The alignment positions (230 aa positions in total) were identical between the original and 6-pair alignments. Note that neither 12aa-motif nor 4aa-motif was included in any of 6-pair alignments.

**Figure 3:**
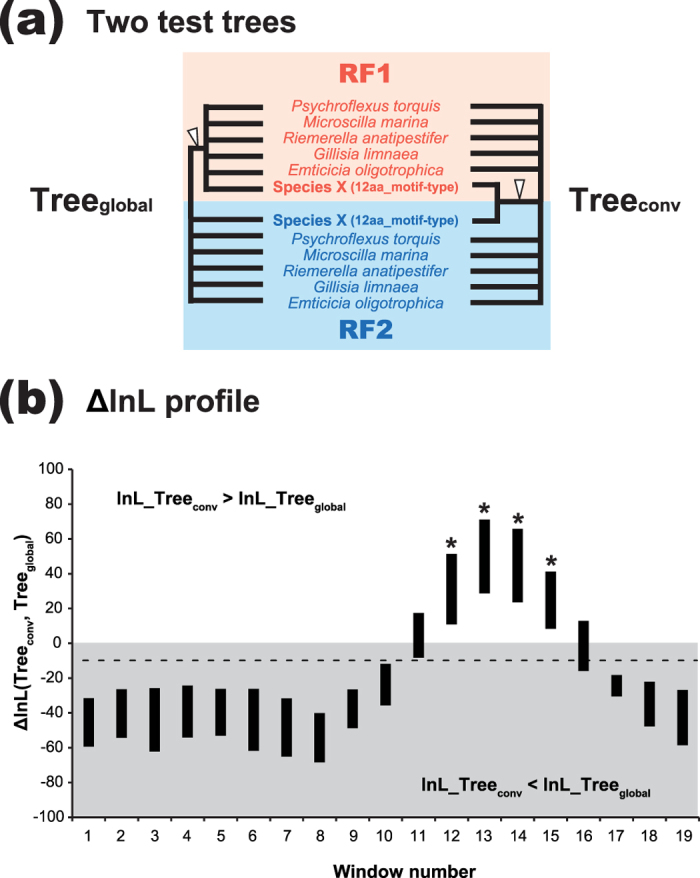
Sliding window analysis for detecting the putative phylogenetic signal of gene conversion between *RF1* and *RF2* genes. (**a**). Schematic presentation of two test trees, Tree_global_ (left) and Tree_conv_, (right) considered in the sliding window (SW) analyses. Tree_global_ represents the ancestral split of RF1 and RF2 affected by no gene conversion. On the other hand, Tree_conv_ bears a clade of the RF1 and RF2 sequences with 12 aa-motif due to gene conversion. As the details of the tree topologies varied depending on individual alignments, all nodes are collapsed in this figure except that representing the RF1-RF2 split in Tree_global_ and that uniting the RF1 and RF2 sequences with 12 aa-motif in Tree_conv_ (highlighted by open arrowheads). (**b**). ∆lnL profile from the SW analyses of 6-pair alignments. The ∆lnL value of an individual sliding window was calculated by subtracting the lnL value of Tree_grobal_ (lnL_Tree_grobal_) from the corresponding value of Tree_conv_ (lnL_Tree_conv_). The SW analyses of 50 alignments generated 50 sets of ∆lnL values. For each window, the range of the 50 ∆lnL values is indicated as a bar. The broken line indicates the estimate of the 0.99^th^ quantile (∆lnL = −9.83) of the null distribution obtained from the parametric bootstrap analysis (see Methods for details). Windows including alignment positions 150 and 151, which are −1 and +1 of 4 aa-/12 aa-motif, are highlighted by asterisks.

In the SW analysis, we compared two tree topologies, Tree_global_ and Tree_conv_, which correspond to the RF evolution incorporating no gene conversion and that assuming the gene conversion, respectively. In the former tree, the RF1 and RF2 sequences are separated from each other regardless of motif-type, representing the ancestral separation of RF paralogues (left in Fig. 3A). For each SW analysis, Tree_global_ was fixed to the ML tree inferred from the entire positions in a particular 6-pair alignment. Consequently, a single Tree_global_ was enforced to all windows, but the branch lengths were re-optimized for each window. On the other hand, Tree_conv_ varied amongst the windows—for each window, we heuristically searched for the ML tree under the constraint of 12aa_motif-type RF1 and RF2 sequences being enforced to form a clade, due to gene conversion (right in Fig. 3B). To detect the phylogenetic signal of the potential gene conversion, the log-likelihood of Tree_global_ (lnL_Tree_global_) was subtracted from that of Tree_conv_ (lnL_Tree_conv_) for each window. If no gene conversion occurred, the resultant difference in log-likelihood between the two test trees (ΔlnL) is expected to be negative. On the other hand, if some windows contain the alignment positions involved in gene conversion, the corresponding ΔlnLs are expected to be positive. The window width was set to 50 aa positions, and the windows were advanced along the alignment by increments of 10 aa positions at a time. It should be noted that the SW analyses in this study were designed exclusively to detect the signal of the conversion between the genes encoding 12aa_motif-type RF1 and RF2. In other words, other types of *RF1-RF2* gene conversion, even if exist, were undetectable in this study.

We recovered similar ΔlnL profiles from the SW analyses of 50 different 6-pair alignments (Fig. 3B). Positive ∆lnL values were constantly observed in windows 12–15 (alignment positions 111–190) in all trials. Interestingly, alignment positions 150 and 151, which are −1 and +1 of the motifs, respectively, are included in windows 12–15 (highlighted by asterisks in Fig. 3B). Consequently, these windows contain aa residues adjacent to 12 aa- or 4 aa-motif, albeit the motifs were excluded from the alignments. From the parametric bootstrap test^22^,^23^, we confirmed that the ΔlnL values calculated from windows 11–15 were significantly higher than those calculated from 99% of 2,500 simulated sequence data under the assumption of no *RF1-RF2* gene conversion (*p* < 0.01; broken line in Fig. 3B). These results indicate that the C-terminal portions of 12aa_motif-type RF1 and RF2 share the phylogenetic signal exclusively, as anticipated in our hypothesis assuming *RF1-RF2* gene conversion. We repeated the SW analyses (plus the corresponding parametric bootstrap analyses) by substituting the original set of 4aa_motif-type RF pairs to three different sets, such as (i) *Bizionia argentinensis, Emticicia oligotrophica*, *Joostella marina*, *Niabella soli*, and *Cyclobacterium marinum*, (ii) *Runella slithyformis*, *Marivirga tractuosa*, *Dyadobacter fermentans*, *Chitinophaga pinensis*, and *Flavobacterium columnare*, and (iii) *Myroides odoratus*, *Niastella koreensis*, *Psychroflexus torquis*, *Solitalea canadensis*, and *Cellulophaga algicola*. Although not shown here, the sampling of 4aa_motif-type RF pairs in a 6-pair alignment appeared to have little impact on the results from the SW analyses. Consequently, we conclude that the RF paralogues in members of Bacteroidia have shared 12 aa-motif due to gene conversion.

The gene conversion between the RF paralogues is supposed to spoil neither function nor tertiary structure of RFs, because the two protein factors are indispensable for translation termination. To evaluate the assumption above, we need to predict precisely the ‘GC-region,’ which was transplanted from one of the RF paralogues to the other. The SW analyses are useful to survey the phylogenetic signal of *RF1-RF2* gene conversion, but can provide only a rough idea for the GC-region. We here predicted the precise boundaries of the GC-region, and subsequently mapped the putative GC-region on the tertiary structures of RF1 and RF2^30^. In addition, by defining the precise GC-region, we can assess strictly the discrepancy of phylogenetic signal between the entire alignment and the GC-region, which is essential to determine the ‘direction’ of *RF1-RF2* gene conversion (see the next section for the details).

**Figure 4:**
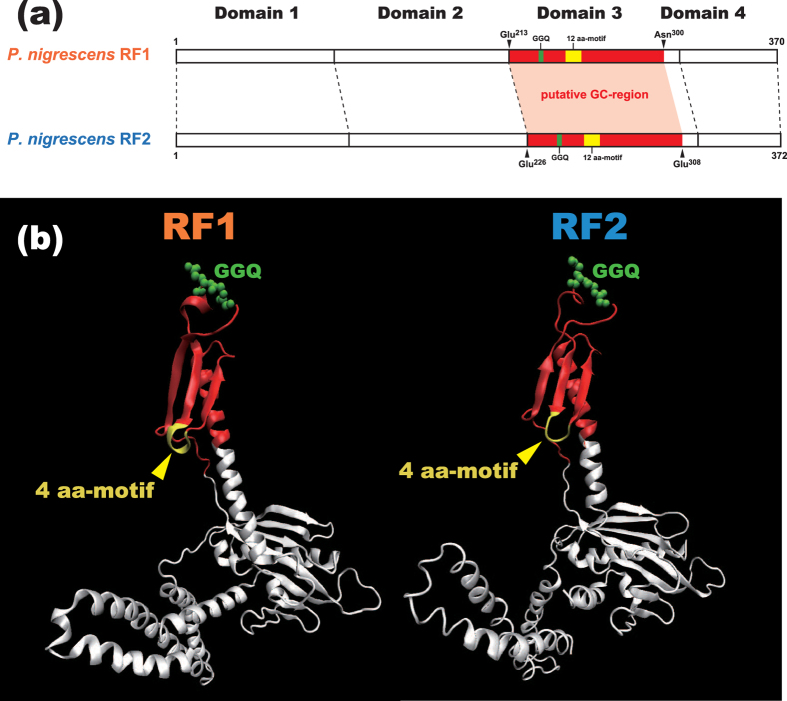
Putative ‘GC-region’ experienced *RF1-RF2* gene conversion. (**a**). Putative GC-region in the primary structures of RF1 and RF2. The GC-region was predicted to start from Glu^213^ to Asn^300^, and from Glu^226^ to Glu^308^ in *Prevotella nigrescens* RF1 and RF2, respectively (highlighted in red). The putative GC-region appeared to occupy a large portion of domain 3, encompassing GGQ motif (green) and 12 aa-motif (yellow). (**b**). Putative GC-region in the tertiary structures of RF1 and RF2. The portions of the tertiary structures of *Thermus thermophilus* RF1 (left) and RF2 (right) (RCSB Protein Data Bank IDs 3MR8 and 2X9R), which correspond to the putative GC-region in the *P. nigrescens* proteins (see above), are colored in red. GGQ motif is presented in the ball-and-stick model (green). 4 aa-motifs in the *T. thermophilius* proteins are colored in yellow.

The boundaries of the GC-region was estimated by subjecting a 6-pair alignment containing a 12aa_motif-type RF pair of *Prevotella nigrescens*, and 4aa_motif-type RF pairs of *Psychroflexus torquis, Microscilla marina, Gillisia limnaea, Emticicia oligotrophica,* and *Riemerella anatipestifer* to a corrected *t* statistic method^24^. As the result, alignment positions 116–186 were defined as the most significant patch of the alignment positions preferring Tree_conv_ over Tree_global_ (i.e. the putative GC-region). The putative GC-region ranges from Glu^213^ to Asn^300^ and from Glu^226^ to Glu^308^ in *P. nigrescens* RF1 and RF2, respectively (GenBank accession nos. EGQ17478.1 and EGQ14454.1). Importantly, the putative GC-region (colored in red in Fig. 4A) appeared to occupy most of RF domain 3 encompassing 12 aa-motif (colored in yellow in Fig. 4A). During translation termination process, domain 3 in both RF1 and RF2 modulate the ribosome to hydrolyse peptidyl-tRNA at the A site^12^. In the tertiary structures of *Thermus thermophiles* RF1 and RF2^30^, 4 aa-motif corresponds to the region connecting two β-sheets in domain 3 (the motif is colored in yellow in Fig. 4B). The acquisition of 12 aa-motif seemingly expanded a loop in RF domain 3, and unlikely had a severe impact on the structure or function of RF proteins (Fig. 4B).

### Single, ancestral *RF2*-to-*RF1* gene conversion followed by multiple motif reversions

**Figure 5:**
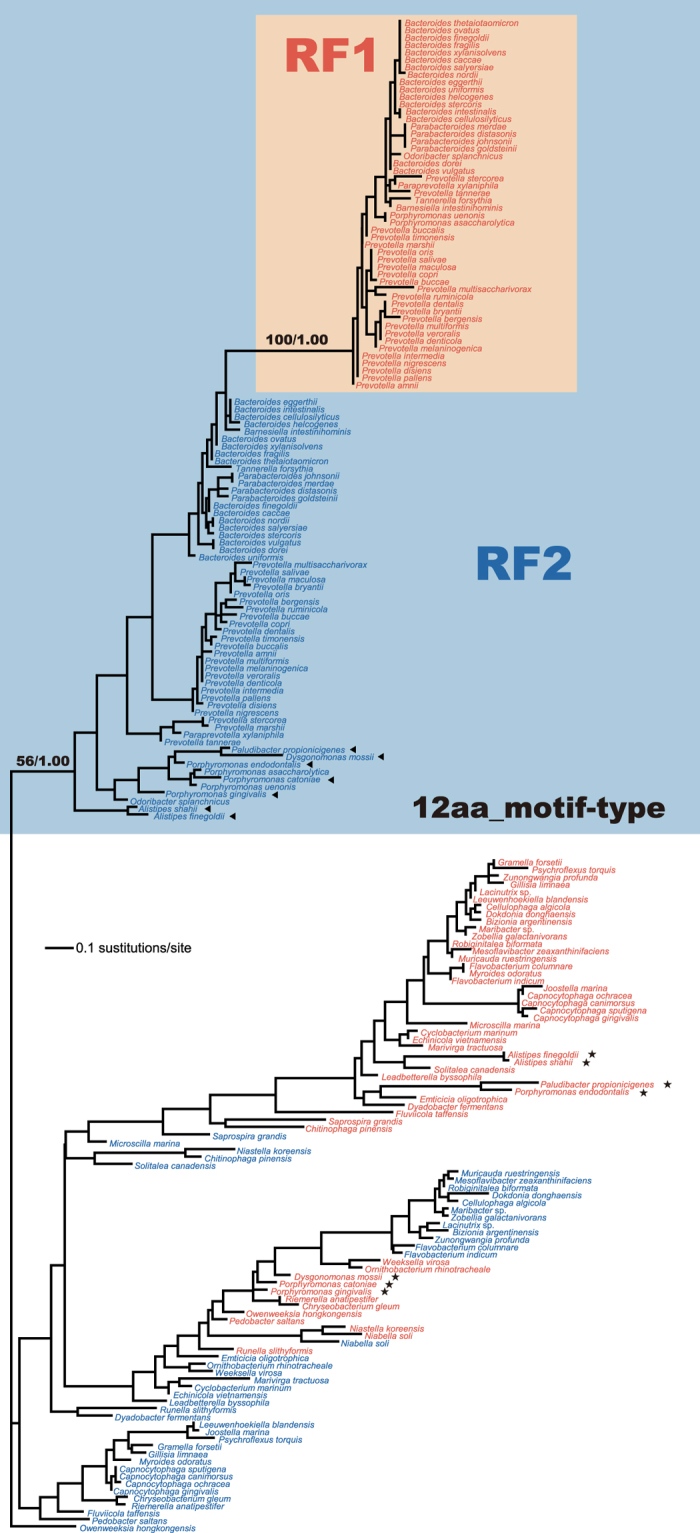
Maximum-likelihood phylogeny inferred from the putative GC-region in RF1 and RF2. The maximum-likelihood (ML) tree was inferred from the putative GC-region (71 amino acid positions) in both RF1 and RF2 sequences of 99 members of Bacteroidetes. Although not shown here, the tree topology inferred by Bayesian method was essentially same as the ML tree. Only ML bootstrap values and Bayesian posterior probabilities for the key nodes are indicated. RF1 and RF2 sequence names are colored in red and blues, respectively. 12aa_motif-type RF1/RF2 sequences are shaded in orange and blue, respectively. The RF1 and RF2 sequences of *Alistipes shahii*, *A. finegoldii*, *Dysgonomonas mossii*, *Paludibacter propionicigenes*, *Porphyromonas* (*Po*) *gingivalis*, *Po. cateniae*, and *Po. endodontalis* are highlighted by stars and arrowheads, respectively.

**Figure 6:**
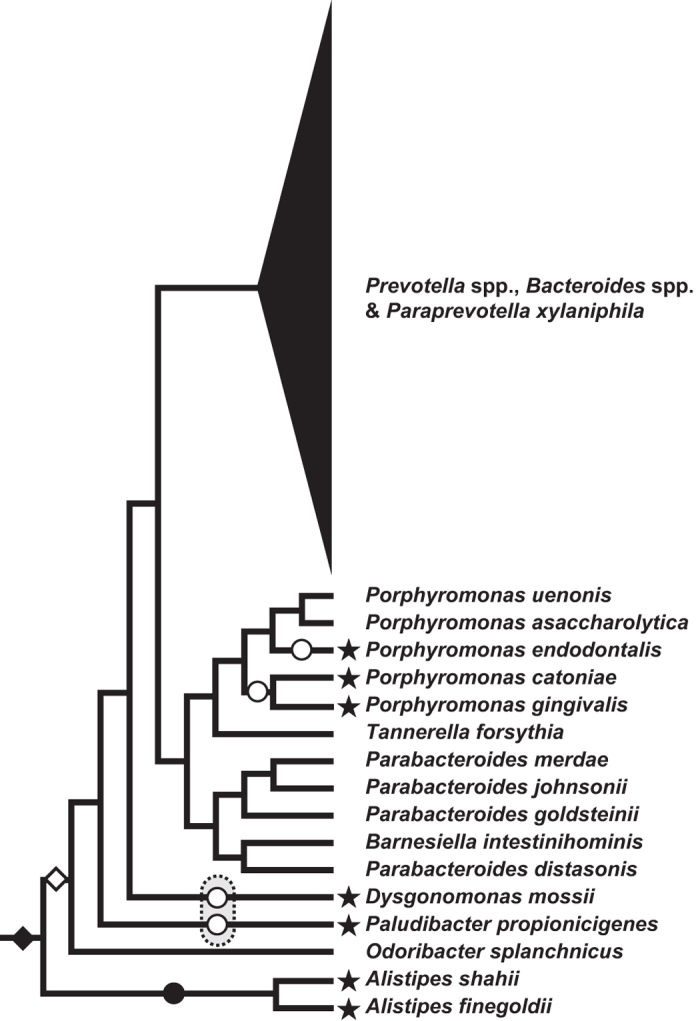
Single gain of 12 aa-motif followed by multiple ‘motif reversions’ in RF1 during the evolution of Bacteroidia. We schematically redrew the rRNA-based organismal phylogeny of Bacteroidia (Fig. 2), and mapped the gain and losses of 12 aa-motif in RF1. The members of Bacteroidia possessing RF1 sequences with 4 aa-motif (4aa_motif-type) are highlighted by stars. We here propose the scenario assuming the first RF1 with 12 aa-motif emerged in the common ancestor of all extant members of Bacteroidia except *Alistipes* spp. (open diamond). This scenario assumes the RF1 sequences of *A. shahii* and *A. finegoldii* as 4aa_motif-type primarily, and reversion from 12 aa-motif to 4 aa-motif (motif reversion) took place independently for four times (open circles). Although not recovered in the ML tree, an alternative tree bearing the monophyly of *Dysgonomonas mossii* and *Paludibacter propionicigenes* failed to be rejected (data not shown). If *D. mossii* and *P. propionicigenes* group together in the organismal phylogeny, motif reversion could have occurred prior to the separation of the two species (two open circles are surrounded by dotted line), reducing the number of motif reversion from four to three. Alternatively, the origin of 12 aa-motif can be pushed back to the common ancestor of all members of Bacteroidia examined here, including *Alistipes* spp. (closed diamond). If so, an additional motif reversion requires on the branch leading to the *Alistipes* clade (closed circle).

The results from the SW analyses clearly suggest that the RF paralogues in Bacteroidia have shared 12 aa-motif due to gene conversion. In this section, we discuss the timing and direction of the gene conversion based on a phylogenetic analysis of the putative GC-region in the alignment of RF1 and RF2 sequences sampled from the 99 members of the Bacteroidetes (71 aa positions). Importantly, both ML and Bayesian analyses successfully recovered all the 12aa_motif-type RF1 sequences as a highly supported clade, and this ‘RF1’ clade as a whole was placed within the radiation of 12aa_motif-type RF2 sequences (Fig. 5). Firstly, we concluded that a *RF1* gene received the *RF2* gene fragment corresponding to the C-terminal portion (including 12 aa-motif), as all of 12aa_motif-type RF1 sequences were recovered as a part of the 12aa_motif-type RF2 clade (Fig. 5). Secondly, the single ‘*RF2*-to-*RF1*’ gene conversion occurred early in the evolution of Bacteroidia, as all of 12aa_motif-type RF1 sequences grouped together in the ML tree (Fig. 5). Combining the points discussed above, we here propose the single *RF2*-to-*RF1* gene conversion in the common ancestor of all members of Bacteroidia except *Alistipes* spp., which were the earliest branching taxa in the rRNA phylogeny (highlighted by an open diamond in Fig. 6; note that we discuss the alternative timing for gene conversion below).

Amongst the 50 members of Bacteroidia examined in this study, *Alistipes* spp., *Paludibacter* (*Pa*) *propionicigenes*, *Dysgonomonas mossii*, *Porphyromonas* (*Po*) *gingivalis*, *Po. cateniae*, and *Po. endodontalis* possess 4aa_motif-type RF1 (open squares in Fig. 2; see also Fig. 1A). Interestingly, in the rRNA phylogeny, *Po. endodontalis* with 4aa_motif-type RF1 showed a close affinity to *Po. uenonis* and *Po. asaccharolytica* with 12aa_motif-type RF1 with a MLBP of 100% and a BPP of 1.00, rather than any species with 4aa_motif-type RF1 (Fig. 2). Furthermore, *Po. endodontalis* and *Po. gingivalis*/*Po. cateniae* were found to be remote from *D. mossii* and *Pa. propionicgenes* in the rRNA phylogeny (Fig. 2). Thus, the RF1 evolution in Bacteroidia demands multiple reversions from 12aa_motif-type to 4aa_motif-type. Based on the Bacteroidia clade in Fig. 2, we here propose that the motif reversion of RF1 occurred independently in (a) *Po. endodontalis*, (b) the common ancestor of *Po. gingivalis* and *Po. cateniae*, (c) *D. mossii*, and (d) *Pa. propionicgenes* (highlighted by open circles in Fig. 6, which is a schematic version of the Bacteroidia clade in Fig. 2). Nevertheless, the relationship between *D. mossii* and *Pa. propionicgenes* was not resolved well in the rRNA phylogeny (Fig. 2). If the two species share a common ancestry by excluding other members of Bacteroidia, the number of independent motif reversions decreases from four to three (Fig. 6).

The putative timing of the *RF2*-to-*RF1* gene conversion discussed above assumes the RF1s of the earliest branching taxa, *Alistipes* spp., are primarily 4aa_motif-type (open diamond in Fig. 6). Nevertheless, as we assume multiple independent motif reversions in the RF1 evolution of Bacteroidia (see above), we cannot exclude the possibility of the secondary loss of 12 aa-motif in *Alistipes* RF1s (filled circle in Fig. 6). This alternative scenario assumes the *RF2*-to-*RF1* gene conversion prior to the divergence of *Alistipes* spp. (filled diamond in Fig. 6). The two scenarios for the timing of the gene conversion are needed to investigate early-branching members of Bacteroidia, as well as the motif in their RF1s, if exist.

Reversion from 12 aa-motif to 4 aa-motif in RF1 was unlikely achieved by shortening of 12 aa-motif or *de novo* re-creation of 4 aa-motif. If a certain 12aa_motif-type RF1 experienced either of the two processes mentioned above, the GC-region encompassing a ‘modified’ 12 aa-motif should retain the original phylogenetic affinity. Nevertheless, the phylogeny of the putative GC-region supported neither of the two possibilities mentioned above, as none of 4aa_motif-type RF1 sequences of the seven species belonging to Bacteroida participated in the clade of 12aa_motif-type RF1 sequences (Fig. 5; 4aa_motfi-type RF1 sequences of interest are highlighted by stars). Interestingly, the indogenous gene encoding 12aa_motif-type RF2 (highlighted by arrowheads in Fig. 5) could not be the source of 4 aa-motif in the genomes of the seven species. Thus, the motif reversion demands the conversion between the gene encoding the indogenous RF1 (presumably of 12aa_motif-type) and a laterally transferred gene encoding a 4aa_motif-type RF1/2. Unfortunately, the precise origins of the reverted 4 aa-motifs are difficult to retrace due to lack of resolution in the phylogenetic analyses of the putative GC-region (Fig. 5).

### *RF1*-to-*RF2* gene conversion in Chloroflexi

**Figure 7:**
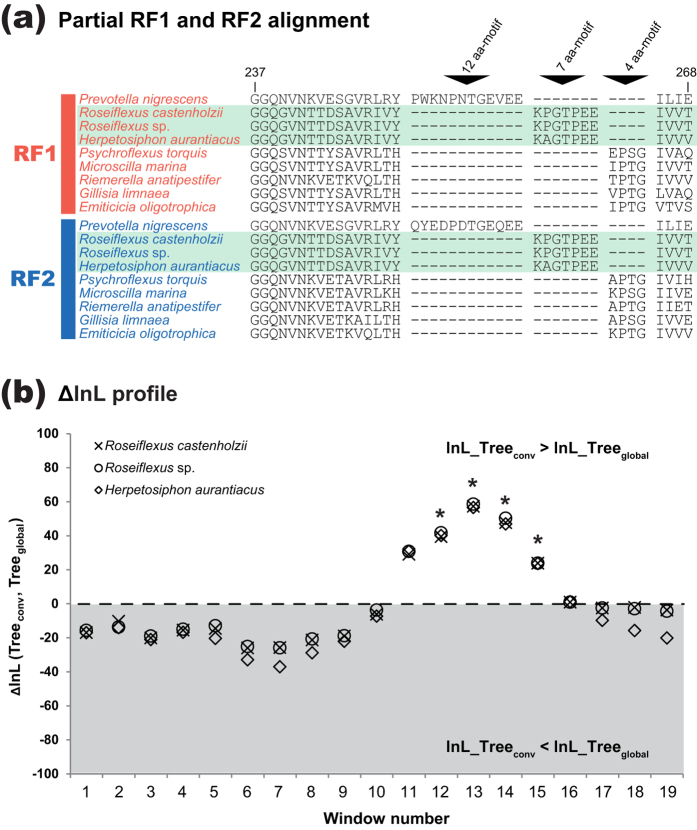
*RF1-RF2* gene conversion in the evolution of Chloroflexi. (**a**). ‘7 aa-motif’ shared between RF1 and RF2 in three members of Chloroflexi. We aligned the RF1 and RF2 sequences of 9 species—six members of Bacteroidetes (*Prevotella nigrescens*, *Psychroflexus torquis*, *Microscilla marina*, *Riemerella anatipestifer*, *Gillisia limnaea*, and *Emticicia oligotrophica*), and three members of Chloroflexi (*Roseiflexus castenholzii*, *Roseiflexus* sp., and *Herpetosiphon aurantiacus*). We here present a portion of the RF1 and RF2 sequences corresponding to amino acid (aa) residues 237–268 in *P. nigrescens* 12aa_motif-type RF1 (GenBank accession no. EGQ17478.1). The RF1 and RF2 sequences of *Roseiflexus* spp. and *H. aurantiacus* are shaded in green. (**b**). ∆lnL profiles from the sliding window (SW) analyses of three ‘6-pair’ alignments. The details of the alignments were described in the main text. Note that neither 4 aa-motif nor 7 aa-motif was remained in 6-pair alignments. The broken line indicates the estimate of the 0.99th quantile (∆lnLs = −0.1) of the null distribution obtained from the parametric bootstrap analysis. The details of this plot are same as described in the legend of Fig. 3B.

The same procedures described above identified an additional case of the putative *RF1-RF2* gene conversion in the evolution of the phylum Chloroflexi. We found a unique ‘7 aa-motif,’ which is shared between RF1 and RF2 of three members of Chloroflexi, *Roseiflexus castenholzii, Roseiflexus* sp., and *Herpetosiphon aurantiacus*. The position of 7 aa-motif is seemingly homologous to those of 4 aa- and 12 aa-motifs, but the three motifs are clearly distinct from each other (Fig. 7A). Thus, we conclude that 7 aa-motif and 12 aa-motif were emerged separately in the RF evolution. To assess whether 7 aa-motif was shared between RF paralogues *via* gene conversion, we analyzed three 6-pair alignments (230 aa positions), which contain a pair of the RF1 and RF2 sequences with 7 aa-motif of *R. castenholzii, Roseiflexus* sp. or *H. aurantiacus*, and five pairs of the 4aa_motif-type RFs of *P. torquis*, *M. marina, Ri. anatipestifer*, *G. limnaea*, and *E. oligotrophica* belonging to Bacteroidetes. As shown in Fig. 7B, the SW analyses, coupled with the corresponding parametric bootstrap test, detected the signal of gene conversion in windows 11–15 (alignment positions 101–190). The boundary estimation based on a 6-pair alignment containing a pair of 7aa_motif-type RF1 and RF2 of *R. castenholzii* nominated alignment positions 108–207 as the putative GC-region. Intriguingly, the putative GC-region occupies almost entire domain 3, as the gene conversion identified in Bacteroidia (see above). The details of the SW analyses and boundary estimation described in this section were same as those assessing the *RF1-RF2* gene conversion in Bacteroidia (see above).

**Figure 8:**
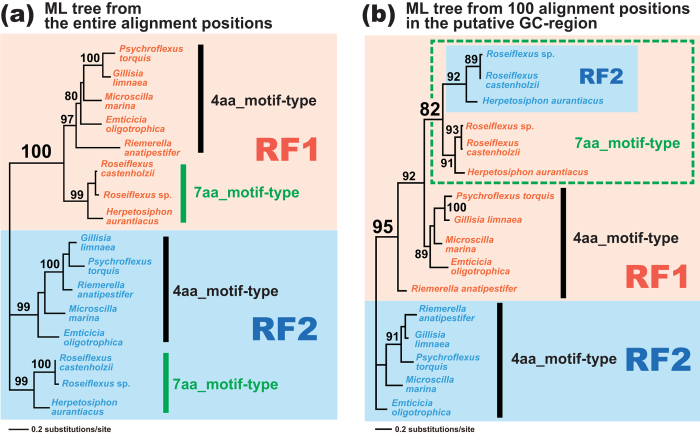
Maximum-likelihood phylogenies inferred from the alignments of the RF1 and RF2 sequences of three members of Chloroflexi and five members of Bacteroidetes. The ‘8-pair’ alignment was subjected to the maximum-likelihood (ML) phylogenetic analyses with the LG + Γ model using RAxML. The details of the phylogenetic analyses are same as those assessing *RF2*-to-*RF1* gene conversion in Bacteroidia described in the main text. ML bootstrap values greater than 80% are shown. (**a**). The ML tree inferred from 230 unambiguously aligned amino acid (aa) positions. The tree topology reflects the ancestral separation between RF1 and RF2. (**b**). The ML tree inferred from 100 unambiguously aligned aa positions in the putative GC-region. The second ML analysis placed ‘7aa_motif-type’ RF2 sequences within the RF1 clade with a specific affinity to those with 7 aa-motif.

We prepared an alignment comprising 8 pairs of RF1 and RF2 sequences sampled from the three members of Chloroflexi and the five members of Bacteroidetes (see above). In the ML tree inferred from the entire ‘8-pair’ alignment including 230 aa positions, the RF1 and RF2 sequences were split with a MLBP of 100% (Fig. 8A). On the other hand, the ML phylogenetic analysis of the putative GC-region (alignment positions 108–207) recovered an intimate affinity of 7aa_motif-type RF2 sequences to the 7aa_motif-type RF1 sequences (boxed by green dotted lines in Fig. 8B), being separated from 4aa_motif-type RF2 sequences in the five members of Bacteroidetes. These results indicate that a *RF1* gene fragment encoding domain 3 was pasted over the corresponding region of a *RF2* gene in the common ancestor of the three members of Chloroflexi (i.e. *RF1*-to-*RF2* gene conversion).

## Conclusion

The present study clearly shows that *RF1-RF2* gene conversion occurred separately in two distinct bacterial lineages (the class Bacteroidia and the phylum Chloroflexi). We regard that gene conversion was functionally neutral in both cases, as the putative GC-regions cover almost entire domain 3, which carries out the identical task between RF paralogues. Intriguingly, the gene fragment encoding RF2 domain 3 was transplanted to the *RF1* gene (i.e. *RF2*-to-*RF1* gene conversion) in the first case, while the *RF2* gene received a *RF1* gene fragment (i.e. *RF1*-to-*RF2* gene conversion) in the second case. The data presented in this study suggest that domain 3 is interchangeable between RF paralogues, and we suspect that many cases of *RF1*-*RF2* gene conversion (particularly the region corresponding to domain 3) have been overlooked in public sequence databases. In this study we customized the settings of the SW analysis based on a priori idea of which RF1 and RF2 sequences with 12 aa-motif underwent gene conversion. However, many of the gene conversion events unlikely associate with informational sequence motifs, and the SW analysis under the same settings as those in this study is not suitable for broad surveys of *RF1*-*RF2* gene conversion in phylogenetically diverse bacteria. For future studies, we need to invent new methods and/or strategies build on robust statistical frameworks, particularly those require no priori assumption on which pair of genes converged with one another.

## Methods

### Phylogenetic analyses

We retrieved the aa sequences of RF1 and RF2, and the nucleotide (nt) sequences of 16S and 23S ribosomal RNA (rRNA) genes, in the genome of 99 taxa belonging to the phylum Bacteroidetes from GenBank database. The retrieved RF1 and RF2 aa sequences are aligned into a single alignment using MAFFT^31^, followed by manual refinement. After the exclusion of ambiguously aligned positions, 230 aa positions were remained in the final RF alignment. The 16S and 23S rRNA nt sequences were separately aligned as described above, and then concatenated into a single alignment. The final rRNA alignment includes unambiguously aligned 3,729 nt positions.

The RF alignment was subjected to both ML and Bayesian phylogenetic analyses. The ML analyses were conducted using RAxML ver. 8.0^32^ under the LG model^33^ incorporating among-site rate variation (ASRV)^34^ approximated with a discrete gamma distribution with four categories (LG + Γ model). The ML tree was selected from the heuristic tree search initiated from 20 randomized stepwise addition parsimony trees. In ML bootstrap analyses (100 replicates), a single tree search per replicate was performed. Bayesian analyses under the LG + Γ model were also conducted using MrBayes 3.2.1^35^. Eight parallel Metropolis-coupled Markov chain Monte Carlo runs, each consisting of one cold and three heated chains with a chain temperature of 0.2, were run for 5,000,000 generations. Log-likelihood scores and trees with branch lengths were sampled at every 1,000 generations. The first 1,250,000 generations were excluded as burn-in, and the remaining trees were summarized to obtain Bayesian posterior probabilities.

The rRNA alignment was subjected to both ML and Bayesian phylogenetic analyses as described above, except the nt substitutions were modelled under the general-time-reversible model^36^ incorporating ASRV approximated with a discrete gamma distribution with four categories (GTR + Γ model). In the alignment, G + C content varied from 49.2 to 59.5%. The impact of the variation in G + C content across a tree on tree reconstruction was evaluated by the additional ML analyses described below. We estimated the 95% confidence interval of the G + C content for each sequence based on the 3,729 nt positions, and surveyed the sequences of which G + C contents significantly depart from the average G + C content calculated from the 99 sequences. Then we modified the original rRNA alignment by removing the sequences with significantly high or low G + C content (Note that the rRNA sequences of the members of Bacteroidia possessing 4aa_mtif-type RF1 were retained in the second alignment, regardless of their G + C contents). The second alignment was subjected to the ML analysis under the GTR + Γ model as described above. In addition, we recoded four nucleotide characters (A, C, G, and T) into purine (R; A or G) and pyrimidine (Y; C or T) in the original alignment, as this ‘RY-coding’ procedure were known to cancel or reduce the artifactual impact of the variation in G + C content in both empirical and simulated nt data on tree reconstruction^28^,^29^,^37^. The resultant ‘RY-coding’ alignment was subjected to the ML analysis with the model of Cavender and Felsenstein for two-state characters^38^ incorporating ASRV approximated with a discrete gamma distribution with four categories. We used RAxML for the ML analyses of the rRNA alignments comprising four nucleotide characters, while PhyML ver. 3.0^39^ was used for the ML analysis of the RY-recoded alignment.

### Sliding window analyses

We generated ‘6-pair’ alignments from the RF alignment to survey the potential signal of the conversion between *RF1* and *RF2* genes. The alignment positions (230 aa positions) in 6-pair alignments were identical to those in the RF alignment. Each 6-pair alignment contained a pair of the 12aa_motif-type RF1 and RF2 sequences and five pairs of the 4aa_motif-type RF1 and RF2 sequences, which were sampled from five species in Bacteroidetes. The detailed sequence sampling in these alignments was described in Results and Discussion. We preliminary subjected ‘4-pair,’ ‘8-pair,’ and ‘10-pair’ alignments, which comprised a single pair of 12aa_motif-type RF1 and RF2 plus 3, 7, and 9 pairs of 4aa_motif-type RF1 and RF2, respectively, to the SW analyses (Fig. S3; see below for the details of the SW analyses). The signal of *RF1-RF2* gene conversion (GC-signal) in windows 12–15 appeared to be less conspicuous in the 4-pair alignment-based analysis than the 6-pair alignment-based analysis (Compare the plot in pink with that in green in Fig. S3). On the other hand, the 10-pair alignment-based analysis was seemingly more sensitive to ‘non GC-signal’ in the N-terminal region (windows 1–10), which were unrelated to the *RF1-RF2* gene conversion, than the 6-pair alignment-based analysis (Compare the plot in blue with that in green in Fig. S3). The GC-signal in windows 12–15 from the 8-pair alignment-based analysis appeared to be conspicuous as that from the 6-pair alignment-based analysis, whereas the aforementioned analysis was more sensitive to non GC-signal in windows 2–6 than the undermentioned analysis (Compare the plot in purple with that in green in Fig. S3). Considering the balance between the sensitivity to the GC-signal and the insensitivity to non GC-signal, we decided to subject 6-pair alignments to the main SW analyses in the current study.

We subjected all 6-pair alignments to the SW analysis^22^,^23^,^24^. For each window, we calculated the lnL of the tree assuming no gene conversion (Tree_global_), and that of the tree affected by gene conversion (Tree_conv_), and then subtracted the former value from the latter value (see Results and Discussion for the details). Statistical significance of the difference between the two lnL values (ΔlnL) was assessed by a parametric bootstrap test^22^,^23^. Seq-Gen version 1.3.3^40^ was used to simulate 50 replicates with 230 aa positions over the ML tree, whose topology and branch lengths were inferred from each of the 50 6-pair alignments, respectively. Of note, all ML trees inferred from 230 aa positions of 50 6-pair alignments recovered the split of RF1 and RF2 sequences. The model parameters for sequence simulation were estimated from the original datasets. We subjected the simulated datasets (2,500 in total) to the SW analysis to obtain the null distribution of the ΔlnL values and set the critical value for a 0.01-level test. We applied RAxML 8.0 with the LG + Γ model for the SW analyses of the original 6-pair alignments. We used the WAG^41^ + Γ model for both sequence simulation and SW analyses based on the simulated sequence data, as LG model is not implemented in Seq-Gen.

The same procedure described above was applied the analyses with the 7aa_motif-type RF1 and RF2 sequences of three members of Chloroflexi (see Results and Discussion).

### Boundary estimation for the region underwent gene conversion

We estimated the precise boundaries of the GC-region by a corrected *t* statistic method developed in Inagaki, Susko, and Roger. 2006^24^. A single 6-pair alignment including the 12aa_motif-type RF1 and RF2 sequences of *Prevotella nigrescens* was subjected to this test. For the boundary estimation, we inferred Tree_global_ and Tree_conv_ from the entire 6-pair alignment positions, and site-wise log-likelihoods (site-lnLs) were separately calculated over the two test trees. Then, for all possible windows, of which size ranged from 30 to 90 aa, we calculated *t* statistics for the test whether the mean site-lnL difference (Δsite-lnL) between the two test trees within a window is same as the corresponding mean Δsite-lnL outside the window. In addition, to adjust for potential window size biases, a *P* value was calculated for each window with a given window width: 10,000 permuted site-lnL datasets for a given window width were subjected to calculate the largest *t* statistics over all possible windows. The *P* value was then calculated as the proportion of test statistics from permuted datasets that were larger than the observed value. Finally, a window which has the largest *t* statistic with a significantly small *P* value (*p* < 0.01) among all possible windows and window widths examined was regarded as the GC-region.

The same procedure described above was applied to the analysis with a single 6-pair alignment including the 7aa_motif-type RF1 and RF2 sequences of *R. castenholzii* and the 4aa_motif-type RFs of five species of Bacteroidetes (*P. torquis*, *M. marina, Ri. anatipestifer*, *G. limnaea*, and *E. oligotrophica*).

### Molecular visualization

The tertiary structures of RF1 and RF2 of *Thermus thermophiles* (RCSB Protein Data Bank IDs 3MR8 and 2X9R, respectively), which reside in the ribosome as a part of the release complex, were visualized using VMD 1.9.1^42^. In this work, the four domains in RF1/2 are defined as per Korostelev (2011)^13^.

## Additional Information

**How to cite this article**: Ishikawa, S. A. *et al.* Multiple conversion between the genes encoding bacterial class-I release factors. *Sci. Rep.* **5**, 12406; doi: 10.1038/srep12406 (2015).

## Supplementary Material

Supplementary materials
