# Supplementary material for: Multiple conversion between the genes encoding bacterial class-I release factors
Source: Sci Rep. 2015 Aug 10;5:12406. doi: 10.1038/srep12406 (PMC4530459; doi:10.1038/srep12406)
Supplement: Supplementary materials [file srep12406-s1.pdf]

Supplementary information

Multiple conversion between the genes encoding bacterial class-I release factor.

Sohta A. Ishikawa[1,2,†], Ryoma Kamikawa[3,4], Yuji Inagaki[1,2]

[1] Graduate School of Life and Environmental Sciences, University of Tsukuba, Tsukuba, Ibaraki, Japan

[2] Center for Computational Sciences, University of Tsukuba, Tsukuba, Ibaraki, Japan

[3] Graduate School of Human and Environmental Studies, Kyoto University, Kyoto, Kyoto, Japan

[4] Graduate School of Global Environmental Studies, Kyoto University, Kyoto, Kyoto, Japan

[†] Current Address: Faculty of Life and Environmental Sciences, University of Tsukuba, Tsukuba, Ibaraki, Japan.

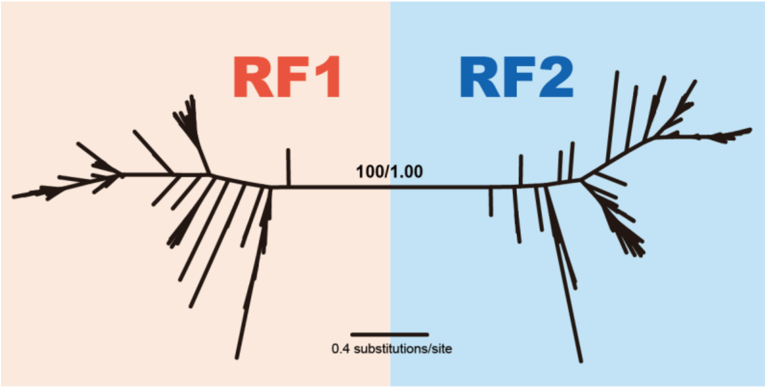

Supplementary Figure S1

Ancestral separation of RF1 and RF2. An alignment comprising the RF1 and RF2 sequences of 99 members belonging to the phylum Bacteroidetes (unambiguously aligned 230 amino acid positions) was analyzed by both maximum-likelihood (ML) and Bayesian methods. As the two methods reconstructed very similar trees, only the ML tree is shown here. The ML bootstrap value and the Bayesian posterior probability are shown only for the split between the RF1 and RF2 subtrees.

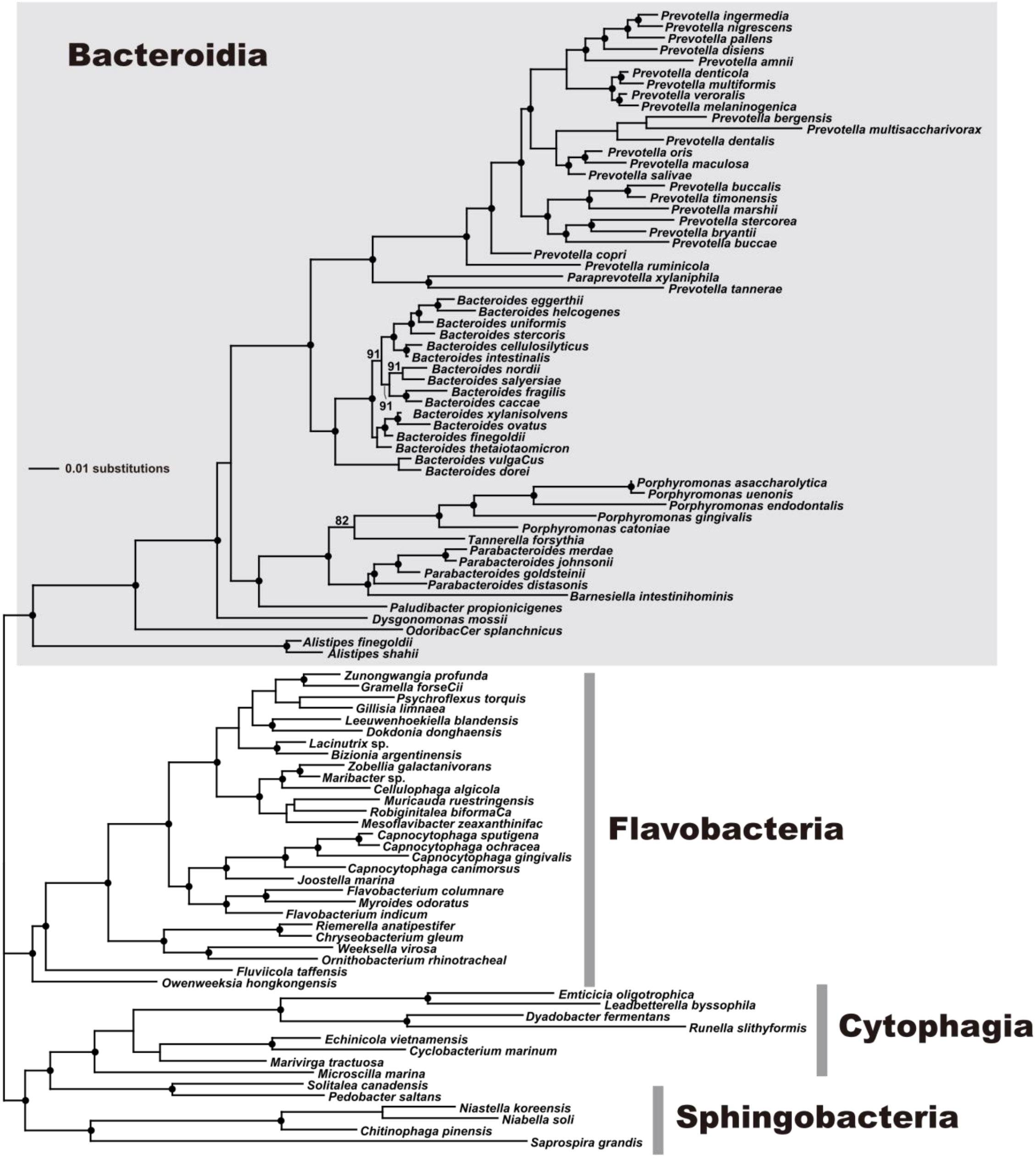

Supplementary Figure S2

Maximum-likelihood tree of Bacteroidetes inferred from a concatenated 16S and 23S ribosomal RNA alignment processed by 'RY-coding' procedure. Four nucleotide characters (A, C, G, and T) in the original rRNA alignment were re-coded into purine (R; A or G) and pyrimidine (Y; C or T), and subjected to the maximum-likelihood method under the model of Cavender and Felsenstein for two-state characters incorporating among-site rate variation approximated with a discrete gamma distribution with four categories. Only ML bootstrap support values (MLBPs) >80% are shown in this figure. Dots correspond to MLBPs of 100%.

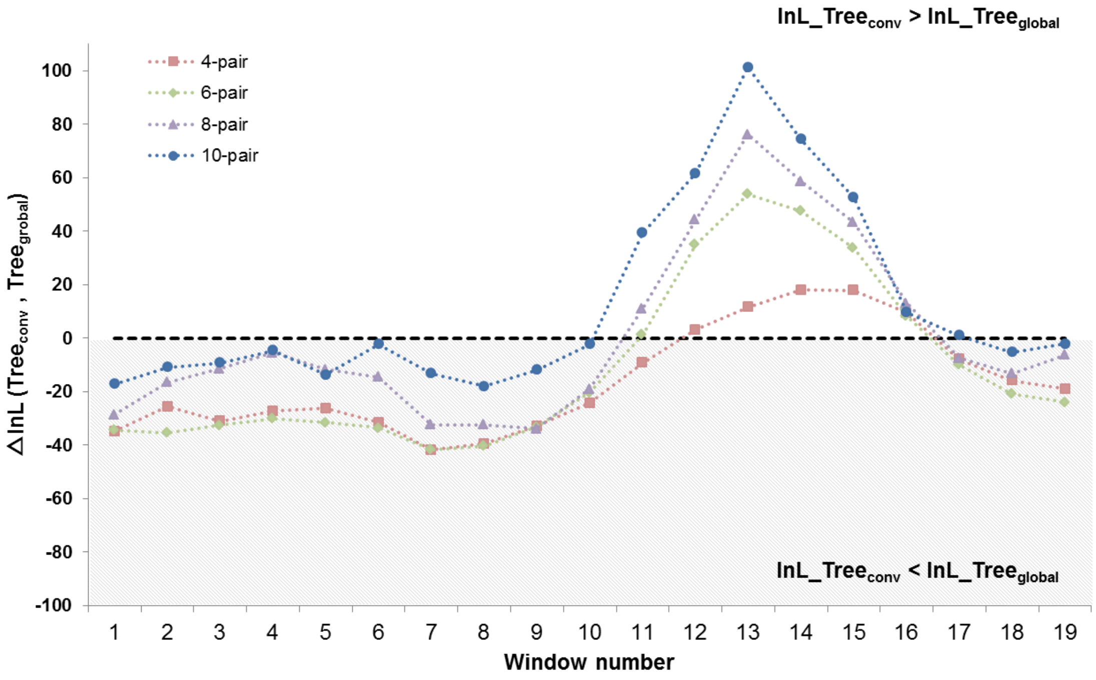

Supplementary Figure S3

Preliminary sliding window analyses of '4-pair,' '6-pair,' '8-pair,' and '10-pair' alignments. To determine the most appropriate sequence numbers in the alignments for the sliding window (SW) analyses, we conducted preliminary SW analyses on four alignments, which were different in the number of 4aa_motif-type RF1 and RF2 sequences: (i) '4-pair' alignment included a single pair of the 12aa_motif-type RF1 and RF2 sequences of *Prevotella nigrescens*, and three pairs of 4aa_motif-type RF1 and RF2 sequences of *Psychroflexus torquis*, *Microscilla marina*, and *Gillisia limnaea*; (ii) '6-pair' alignment was generated by adding the RF1 and RF2 sequences of *Riemerella anatipestifer* and those of *Emticicia oligotrophica* to 4-pair alignment; (iii) '8-pair' alignment was generated by adding the RF1 and RF2 sequences of *Runella slithyformis* and those of *Niastella koreensis* to 6-pair alignment; (iv) '10-pair' alignment was generated by adding the RF1 and RF2 sequences of *Bizionia argentinensis* and those of *Echinicola vietnamensis* to 8-pair alignment. The alignment positions were identical among the four alignments (230 unambiguously aligned aa positions). The details of the SW analyses are same as those described in the main text. The ΔlnL profiles from the SW analyses of 4-pair, 6-pair, 8-pair, and 10-pair alignments were colored in red, green, purple, and blue, respectively. For these preliminary SW analyses, we conducted no parametric bootstrap analysis.
